# Applicants for Membership in the United States Veterinary Medical Association

**Published:** 1892-09

**Authors:** 


### APPLICANTS FOR MEMBERSHIP.

Members knowing any reason why any of the following should not become members of the United States Veterinary Medical Association, will please forward same in writing to the Secretary, before the meeting of the Comitia Minora, September 19th, or appear at the meeting and make statement of grounds for objection :

### HONORARY MEMBERSHIP.

Wm. H. Welch, M.D., Prof. of Pathology Johns Hopkins University. Recommended by A. W. Clement, Rush S. Huidekoper. Endorsed by Maryland State Veterinary Medical Association.

### ACTIVE MEMBERSHIP.

John A. Bell, V.S., Ontario, Watertown, N. Y. Vouchers, Nelson P. Hinkley and John Wende.

Theobald Smith, Ph.B., M.D., Cornell Univ., Albany Med. Coll., 1527 O st., N. W. Washington, D. C. Vouchers, C. B. Michener, and W. L. Williams.

A. M. Farrington, B.V.S., Vet. Dept. Cornell, Dept. of Agriculture, Washington, D. C. Vouchers, C. B. Michener and W. H. Lowe.

Wm. B. Werntz, V.M.D., Vet. Dept. U. of Pa., 4531 Lancaster ave., Phila , Pa. Vouchers, Rush S. Huidekoper and S. E. Weber.

I. N. Krowl, D.V.S., Am. Vet. Coll., Passaic, N. J. Vouchers, J. F. Winchester and Austin Peters.

F. K. Chaffee, D.V.S., Chicago Vet. Coll., Chicago, Ill. Vouchers, A. H. Baker and S. S. Baker.

J. D. Hinebaugh, M.S., V.S., Ontario, Fargo, N. Dakota. Voucher, W. L. Williams.

H. W. Hawley, D.V.S., Chicago, Chicago, Ill. Vouchers, A. H. Baker and S. S. Baker.

G. Allan Jarman, D.V.S., Am. Vet. Coll., Chestertown, Md. Vouchers, A. J. Thompson and J. A. Huhne.

Fred W. Ashe, D.V.S., Chicago, Chicago, Ill. Vouchers, A. H. Baker and S. S. Baker.

John Hall, D.V.S., Am. Vet. Coll., Falls City, Neb. Vouchers, Prof. A. Liautard and John S. Meyer.

T. B. Pote, D.V.S., Montreal, Mt. Vernon, Ind. Voucher, W. L. Williams.

C. Frank Dwinal, D.V.S., Am. Vet. Coll., Mechanics Falls, Me. Voucher, Geo. H. Bailey.

H. H. Choate, D.V.S., Am. Vet. Coll., Lewiston, Maine. Voucher W. J. Coates.

T. Earle Budd, D.V.S., Am. Vet. Coll., Woodbury, N. J. Voucher, E. B. Ackerman.

R. P. Steddom, V.S., Ontario, Galesburg, Ill. Vouchers, C. E Hollingsworth and O. J. Lanigon.

John Forbes, M.R.C.V.S., Glasgow, Scotland, S. Omaha, Neb. Voucher, S. Stewart.

Wm. L. Nunan, D.V.S., Am. Vet. Coll., Lansdowne, Pa. Voucher, W. Horace Hoskins.

R. A. Archibald, D.V.S., Chicago Vet. Coll., Sacramento, Cal. Vouchers, A. H. Baker and R. J. Withers.

John Doris, Jr., D.V.S., Ohio Vet. Coll., 152 Centre ave., Pittsburg, Pa. Vouchers, Jas. A. Waugh and J. C. McNeill.

H. F. Doris, D.V.S., Am. Vet. Coll., 37th st., Pittsburg, Pa. Voucher, W. Horace Hoskins.

Wyatt Johnson, M.D.C.M., McGill Univ. Med. Coll., Montreal, Can. Vouchers, A. W. Clement and J. F. Winchester.

F. A. Nief, B.S.C., Am. Vet. Coll., 921½ Post st., San Francisco, Cal. Vouchers, E. J. Nesbitr, E. B. Ackerman.

Warren Thomas Edwards, V.M.D., Vet. Dept. U. of Pa. Clearfield, Clearfield Co., Pa. Vouchers, W. Horace Hoskins and R. Webster.

Isidore Turcot, V.S., Montreal, Minto, Walsh Co., N. Dak. Voucher, M. A. Piche.

Claude D. Morris, V.S., Ontario, Bath, N. Y. Vouchers, John Wende and Nelson P. Hinkley.

Leonard Pearson, V.M.D., Vet. Dept. U. of Pa., 2200 Pine st., Philadelphia, Pa. Vouchers, W. Horace Hoskins and A. T. Sellers.

Robert Formad, V.M.D., Vet. Dept. U. of Pa., 1008 N. 6th st., Philadelphia, Pa. Voucher, W. Horace Hoskins.

A. Darling, V.D.S., McGill Univ. Med. Coll., 3230 Locust st., St. Louis, Mo. Vouchers, Nelson P. Hinkley and John Wende.

R. A. Dunn, D.V.S., Am. Vet. Coll., Titusville, Pa., Vouchers, A. Liautard and W. Horace Hoskins.

Wm. H. Valway, V.S., N. Y. Vet. Coll., 2698 Broadway, Cleveland, O. Voucher, Wm. R. Howe.

J. M. Chase, V.S., Ontario, Seneca Falls, N. Y. Vouchers, John Wende and Nelson P. Hinkley.

J. D. Robinson, D.V.S., Chicago Vet. Coll., 222 C st., Washington D. C. Vouchers, A. W. Baker and E. S. Walmer.

N. C. Jones, V.S., Ontario, Chillicothe, O. Voucher, Tait Butler.

J. S. Butler, V.S., Ontario, Minneapolis, Minn. Voucher, Tait Butler and C. C. Lyford.

Walter L. Burt, D.V.S , Am. Vt. Coll., 26 Taber av., Providence, R. I. Voucher, W. Horace Hoskins.

Thos. Griffin, M.R.C.V.S., Royal Coll. of Vet. Surgeons, London, Eng., 125 W. 56th st., New York City. Voucher, R. S. Huidekoper.

A. J. Sheldon, D.V.S , Am. Vet. Coll., 141 W. 54th st., New York City. Vouchers, A. Liautard and W. J. Coates.

C. H. Pierce, V.S., Ontario, Minneapolis, Minn. Voucher, Tait Butler and C. C. Lyford.

H. D. Hanson, D.V.S., Am. Vet. Coll., 160 Eldridge st., New York City. Vouchers, A. Liautard and W. J. Coates.

John B. Hopper, D.V.S., Am. Vet. Coll., Ridgewood, N. J. Vouchers, Wm. H. Lowe and A. Liautard.

E. D. Bachman, D.V.S., Am. Vet. Coll., Chester, Orange Co , N. Y. Vouchers, Wm. H. Lowe and A. Liautard.

E. R. Ogden, D.V.S., Am. Vet. Coll., Orange, N. J. Vouchers, Wm. H. Lowe and A. Liautard.

J. Payne Lowe, D.V.S., Am. Vet. Coll., Paterson, N. J. Vouchers, Wm. H. Lowe and A. Liautard.

J. M. Chase, V.S., Ontario, Seneca Falls, N. Y. Vouchers, R. A. McLean and N. P. Hinkley.

Chas. Lintz, V.M.D., U. of Pa., 316 E. Broad st., Chester, Pa. Voucher, W. Horace Hoskins.

E. L. Loblein, D.V.S., Am. Vet. Coll., New Brunswick, N. S. Voucher, Wm. H. Lowe.

J. E. Cloud, D V.S., Chicago Vet. Coll., Richmond, Wayne Co., Ind. Voucher, W. L. Williams.

Lee Hoover, D.V.S , Chicago Vet. Coll., Richmond, Wayne Co., Ind. Voucher, W. L. Williams.

Richard Ebbitt, M.R.C.V.S , Royal Coll. of Vet. Surgeons, Glasgow, Omaha, Neb. Vouchers, A. H. Baker and S. Stewart.

A. D. McKenney, V.S., Ontario, Ann Arbor, Mich. Voucher, E. A. A. Grange.

James McDonough, D.V.S., Am. Vet. Coll. Montclair, N. J. Voucher, Wm. H. Lowe.

Alex. Burr, M.D.V., Harvard Coll. Brighton, Mass. Vouchers, W. Bryden and L. H. Howard.

